# Characterization of the human N^α^-terminal acetyltransferase B enzymatic complex

**DOI:** 10.1186/1753-6561-3-S6-S4

**Published:** 2009-08-04

**Authors:** Amagoia Ametzazurra, Cristina Gázquez, Marta Lasa, Esther Larrea, Jesús Prieto, Rafael Aldabe

**Affiliations:** 1 Division of Hepatology and Gene Therapy, Center for Applied Medical Research (CIMA), University of Navarra, Pamplona, Spain; 2 Liver Unit, Clínica Universitaria, CIBER-EHD, Pamplona, Spain; 3 Proteomika, S.L. Parque Tecnológico de Zamudio. Edificio 504. 48160 Derio, Spain

## Abstract

**Background:**

Human N^α^-acetyltransferase complex B (hNatB) is integrated by hNaa20p (hNAT5/hNAT3) and hNaa25p (hMDM20) proteins. Previous data have shown that this enzymatic complex is implicated in cell cycle progression and carcinogenesis. In yeast this enzyme acetylates peptides composed by methionine and aspartic acid or glutamic acid in their first two positions respectively and it has been shown the same specificity in human cells.

**Methods:**

We have silenced *hNAA20 *expression in hepatic cell lines using recombinant adenoviruses that express specific siRNAs against this gene and analyzed cell cycle progression and apoptosis induction after this treatment. Immunopurified hNatB enzymatic complexes from human cell lines were used for analyzing hNatB *in vitro *enzymatic activity using as substrate peptides predicted to be acetylated by NatB.

**Results:**

*hNAA20 *silencing in hepatic cell lines reduces cell proliferation in a p53 dependent and independent manner. At the same time this treatment sensitizes the cells to a proapototic stimulus. We have observed that the hNatB complex isolated from human cell lines can acetylate *in vitro *peptides that present an aspartic or glutamic acid in their second position as has been described in yeast.

**Conclusion:**

hNatB enzymatic complex is implicated in cell cycle progression but it exerts its effects through different mechanisms depending on the cellular characteristics. This is achievable because it can acetylate a great number of peptides composed by an aspartic or glutamic acid at their second residue and therefore it can regulate the activity of a great number of proteins.

## Background

Protein modification is a mechanism for regulate and improve proteins function and activity, being N^α^-terminal acetylation one of the most abundant activities. Although the two cotranslational events, cleavage of the initiator methionine and N^α^-acetylation, are common and highly conserved from bacteria to higher eukaryotes, their function it is not well understood [1,2]. Obviously, the removal of the initiator residue allows diversity of aminoterminal sequences and therefore enhances the functional repertoire of the polypeptide.

N^α^-acetyltransferase complexes are composed by one catalytic subunit and one or several auxiliary subunits. NatB enzymatic complex is integrated by the catalytic subunit Naa20p and the auxiliary subunit Naa25p, as has been observed in yeast and human cells [3-5].

In yeast, proteins with Met-Asp-, Met-Glu-, Met-Asn- and Met-Met- amino termini constitute potential NatB substrates. However, whereas Met-Asp- and Met-Glu- termini appear to be acetylated in 100% of the cases, only some of the experimentally investigated Met-Asn- and Met-Met- termini have been found to constitute true NatB targets [6]. In the meantime all mammals proteins with Met-Asn- and Met-Met- termini analyzed present the methionine acetylated and consequently they are considered as NatB substrates [2].

Despite the widespread occurrence of N^α^-terminal acetylation in eukaryotes, the biological relevance of this protein modification has only been deduced for a few substrates, as the tropomyosin and actin. It has been documented that yeast tropomyosin-1 and actin are NatB-mediated acetylated being this modification important for the formation of stable and functional actin cables [3,4]. Tropomyosin activity is also regulated by N-terminal acetylation in fission yeast [7] and N-terminal acetylation of Dictiostelum actin strengthens interaction of actin and myosin [8]. In addition, actin aminoterminal processing is ligated to the acetylation of the initial methionine as the removal of the methionine in class I and class II actins and of the cysteine in class II actins that occurs in an acetylation-dependent manner [9,10]. Hence, a proper N-terminal processing is very important for regulating actin function [11].

Human NatB complex has been recently characterized showing that it is composed at least by the catalytic subunit hNaa20p and the auxiliary subunit hNaa25p [5]. The two main aminoterminal acetyltransferase activities, NatA and NatB, are important for human cell cycle progression [5,12], but unlike NatA, there is not an induction of apoptosis when NatB is inhibited [5,12,13].

We have extended the analysis of NatB function to two human hepatocarcinoma cell lines concluding its implication in cellular proliferation. We have also studied in *vitro *hNatB activity identifying some new *in vitro *substrates and establishing that not all the peptides with Met-Asp- and Met-Glu- amino termini are good *in vitro *hNatB substrates.

## Methods

### hNAA20 and hNAA25 expression plasmids, siRNAs and adenovirus production

*hNAA20-CTAP *expression plasmid was generated excising hNAA20 from the pcDNA3-TOPO-TA-hNAA20 plasmid and inserting it in the plasmid pCTAP (Stratagene, CA, USA). The plasmid pDEST27-NAA25 was obtained from Imagenes (Berlin, Germany).

*hNAA20 *expression in HepG2 and Hep3B cell lines has been inhibited using recombinant adenoviruses that express specific siRNAs. These sequences and adenovirus production have been described previously [12].

### Cell culture and infections with the siRNA expressing adenoviruses

Human Hela, HepG2, Hep3B and 293 cell lines were purchased from the ATCC and 293 Cre4 cell line was provided by Dr Hardy. All the cell lines were cultured in DMEM supplemented with 10% foetal calf serum. Cre4 cells were also supplemented with 500 μg/ml G418. All reagents were from Gibco-BRL (Paisley, UK).

Infections with *hNAA20 *siRNA expressing adenoviruses were performed in HepG2 and Hep3B cells. The day prior to infection 250,000 HepG2 and 150,000 Hep3B cells were plated in a 6 well plate and infection was performed in DMEM-2% foetal calf serum at a multiplicity of infection (MOI) of 10.

### Protein extraction and Western Blot Analysis

To extract protein from cultured cells, the cells were collected in 100 μl of RIPA solution (150 mM NaCl, 50 mM Tris pH 7.5, 0.1% SDS, 1% Triton X-100, 0.5% sodium deoxycholate, 10 mM NaF, 1 mM Na_3_VO_4 _and a protease inhibitor cocktail from Roche). Protein extracts were collected after sonication and centrifugation at 16,000 g for 15 minutes, and supernatants were stored at -80°C.

Cultured cells protein extracts (20 μg) were loaded in SDS polyacrylamide gels and after electrophoresis, the proteins were transferred to nitrocellulose membranes (Bio-Rad Laboratories) and detected by incubation with specific antibodies. Protein bands were visualised using the enhanced chemiluminescence detection system (Perkin Elmer, Boston, Massachusetts, USA) and membranes were autoradiographed.

Antibodies used were: chicken anti-hNAA20 from Genway Biotech (California, USA), goat anti-GST from Abcam (Cambridge, UK), mouse anti-GAPDH from Biogenesis (Bournemouth, UK), rabbit anti-Bcl-2, mouse anti-Mdm2, mouse anti-p53 (DO-1), goat anti-p21(WAF/CIP1)(WAF1/CIP1) from Santa Cruz Biotechnology (Santa Cruz, California, USA), anti-rabbit IgG HRPO from Cell Signalling (Beverly, Massachusetts, USA), anti-goat IgG HRPO and anti-mouse IgG HRPO from Sigma, rabbit anti-IgY from Upstate and rabbit anti-IgY HRPO from Pierce (Illinois, USA).

### Coimmunoprecipitation

GST-hNAA25 and hNAA20-CTAP expressing plasmids were transfected in Hela cells using linear polyethylenimine 25 kDa (Polysciences, Warrington, PA, USA) as described previously [14]. 48 hours after transfection the samples were harvested in RIPA buffer and incubated with anti-Naa20p or anti-GST antibodies to immunoprecipitate the protein complexes.

### N-terminal acetyltransferase assay (NAT)

The NAT assay was performed basically as described previously [15,16] but using hNaa20p or GST-hNaa25p immunoprecipitated complexes that were incubated with 138 μl of 0.2 M K_2_HPO_4 _(pH 8.1), 10 μl of the substrate peptide (0.5 mM) and 1 μCi of [^3^H]acetyl-CoA (99.9 GBq/mmol, GE Healthcare) for 2 hours at 37°C. The samples were centrifuged and the supernatant was incubated with SP-Sepharose (50% in 0.5 M acetic acid) for 10 minutes on a rotor before washing the SP-Sepharose three times with acetic acid 0.5 M and once with methanol. The radioactivity incorporated in the peptides was determined by scintillation counting.

### Cell proliferation assay

HepG2 and Hep3B cells were seeded in 15 mm cover glasses and infected with siRNA expressing adenovirus pAdsiRNA2 or pAdsiRNA350 for 24, 48 and 72 hours, BrdU labeled for 1 hour and processed using the 5-bromo-2'-deoxyuridine Labeling and Detection Kit I (Roche Applied Science, Penzberg, Germany) following the manufacturer's instructions.

### Cell cycle analysis

HepG2 and Hep3B cells were harvested after 72 hours of infection with the adenovirus pAdsiRNA2 or pAdsiRNA350 and processed using the CycleTEST PLUS DNA reagent kit (Becton Dickinson) following the manufacturer's instructions. Flow cytometric analysis of the different samples was performed using BD FACScalibur flow cytometer and DNA content was analyzed using the MODFIT software.

### TUNEL Assay

HepG2 and Hep3B cells were incubated in 15 mm cover glasses and infected with adenovirus pAdsiRNA2 or pAdsiRNA350 for 48 hours prior adding the proteasome inhibitor MG132 (5 μM) (Calbiochem, Darmstadt, Germany) for an additional 8 hours. Cells were fixed in 4% phosphate-buffered paraformaldehyde (pH 7.4). TUNEL assay was performed using the In Situ Cell Death Detection Kit (Roche Applied Science) according to the manufacturer's instructions. Cell apoptosis percentage was obtained as indicated in cell proliferation assay.

## Results and discussion

### hNaa20p implication in cellular proliferation

**Figure 1:**
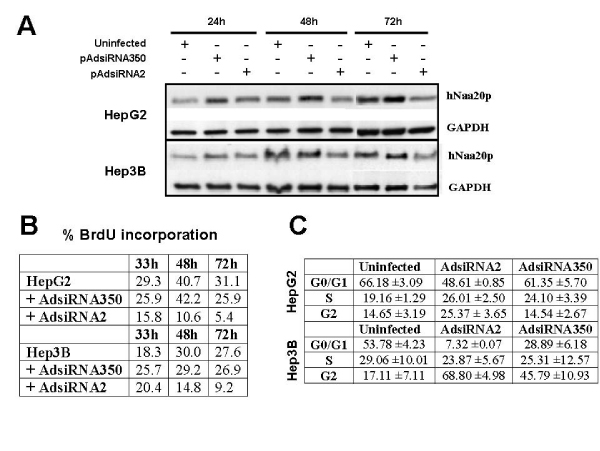
**Effects of siRNA mediated hNAA20 expression inhibition in HepG2 and Hep3B cellular proliferation**. A. Western Blot analysis of hNaa20p in uninfected or infected cells with adenoviruses which express siRNA2 or control siRNA (siRNA350) for 24, 48 and 72 hours. B. Cellular proliferation quantification after siRNA mediated hNAA20 knockdown for 33, 48 and 72 hours measured as the percentage of cells that incorporate BrdU. Representative experiment of at least three independent experiments. C. Cell cycle flow cytometry analysis of control and hNAA20 downregulated expression in HepG2 and Hep3B cells 72 hours after adenovirus infection. The data represent the mean value and standard deviation of the cell percentage present in each phase of the cell cycle from four independent experiments.

It has been previously reported that hNatB inhibition generates a reduction of cellular proliferation [5,12]. To extend this analysis to the effect of hNaa20p inhibition in human hepatocarcinoma cell lines, we performed siRNA mediated hNaa20p downregulation infecting HepG2 and Hep3B cell lines with recombinant adenoviruses that express specific or unrelated *hNAA20 *shRNAs. As it is observed in Figure 1A, 72 hours after viral infection there is close to a 75% decrease of hNaa20p present in HepG2 and Hep3B cells when *hNAA20 *siRNA (pAdsiRNA2) is expressed. Previous data indicate that there is an inhibition of cell cycle progression when hNatB is inhibited. Therefore we measured cellular proliferation after *hNAA20 *knockdown and observed a clear inhibition in both cell lines (Figure 1B). This growth arrest is accompanied with an accumulation of cells in the G2 phase of the cell cycle after a FACS cell analysis (Figure 1C). We have previously observed the same effect in Hela cells, in contrast to the G0/G1 accumulation observed by Starheim *et al *[5] after siRNA mediated hNaa20p downregulation in Hela and CAL-62 cell lines. Interestingly, we observed an increase of the percentage of cells in G2 phase in Hep3B after unrelated siRNA (siRNA350) expression, indicating that adenovirus mediated siRNA expression could be facilitating G2 phase accumulation. In spite of this effect, there is no reduction in the rate of proliferation after siRNA350 expression in Hep3B (Figure 1B). When we have used another h*NAA20 *siRNA or unrelated h*NAA20 *shRNA [12]we have obtained similar results (data not shown).

Thus hNatB inhibition promotes a reduction in cellular proliferation in all cell lines tested, according to the effects of hNatA inhibition [13,17]. It seems therefore that N-terminal acetylation of proteins is an important function for a proper cell growth and division as has been described for protein lysine acetylation/deacetylation [18].

**Figure 2:**
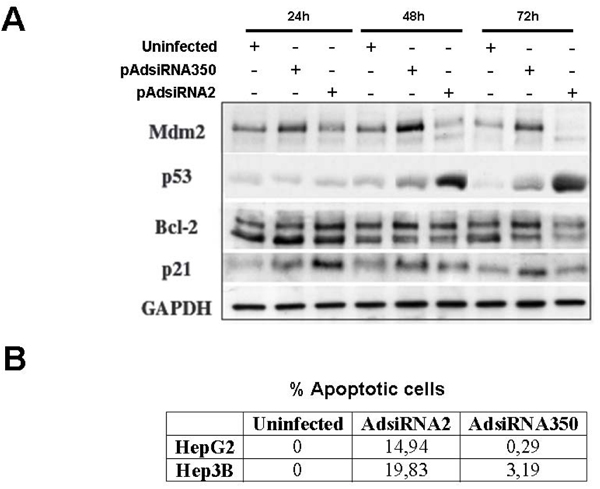
**Effects on HepG2 and Hep3B p53 expression and cell death after siRNA mediated hNAT5/hNAT3 knockdown**. A. Western Blot of Mdm2, p53, Bcl-2 and p21(WAF/CIP1) 24, 48 and 72 hours after infection using GAPDH as loading control. B. Apoptotic cell death quantification using TUNEL technology after hNaa20p expression inhibition for 48 hours and incubated with the proteasome inhibitor MG132 for additional 8 hours. Representative experiment of at least three independent experiments.

The p53 tumour suppressor is a tightly regulated protein that acts by stopping cell-cycle progression or promoting apoptosis when cells encounter stress stimuli such as oncogene activation or DNA damage [19,20]. For that reason we analyzed HepG2 cellular extracts for p53 accumulation, and 48 hours after siRNA expression there was an increase in the amount of p53 present in the cells corresponding with the decrease of Naa20p (Fig 2A), as has been described in Hela cells [12]. As it is shown in Figure 2A, p53 accumulation in HepG2 correlated with a decrease of Mdm2 and not with p53 Ser15 phosphorylation as was described in Hela cells [12]. One of the mediators of the response generated after p53 activation is p21(WAF/CIP1) that is activated in Hela cells but not in HepG2. Hence *hNAA20 *downregulation promotes different stress signals in Hela and HepG2 cells causing growth arrest. But this antiproliferative effect can also be p53 independent because hNaa20p downregulation in Hep3B, a cell line that is p53 deficient [21], induces cell growth arrest too. Consequently, when we compared Hela gene expression profile obtained after *hNAA20 *downregulation with HepG2 and Hep3B most of the genes induced in Hela cells were not upregulated in HepG2 and Hep3B (data not shown). These differences are not restricted to *hNAA20 *inhibition in distinct cell lines because *hNAA25 *repression causes also cell growth arrest, but instead of upregulating p21(WAF/CIP1) expression, as *hNAA20 *knockdown, there is a clear p21(WAF/CIP1) reduction [5]. The different molecular responses generated after hNaa20p downregulation in different cell lines can be a reflection of the high number of hNatB substrates (above 10% of total proteome) that can affect cellular growth according to their acetylation status and the cellular milieu, as it has been described for histone deacetylase inhibitors [18]. But we can not exclude the existence of a common mechanism in all cell lines that we have not deciphered yet.

### hNaa20 knockdown sensitizes the cells to proapoptotic stimuli

In many instances inhibition of cellular proliferation is associated with an increase of apoptosis [22]. Inhibition of NatB activity in Hela cells sensitizes the cells to a proapoptotic stimulus, like the proteasome inhibitor MG132 [12], but it is not associated with an increase of cellular apoptosis. We observed that *hNAA20 *knockdown in HepG2 and Hep3B is not associated with apoptosis induction (data not shown ) but it sensitizes the cells to the proapoptotic treatment with MG132 as it is presented in Figure 2B where there is a clear induction of apoptosis in HepG2 and Hep3B cells that express *hNAA20 *siRNA, siRNA2. This effect correlates in HepG2 with a reduction of BCL2 (Figure 2A), which is an important antiapoptotic molecule [23].

We have observed also that Hep3B cell line is more sensitive to MG132 treatment as Hep3B cells that express the unrelated siRNA present some sensitivity to MG132. This is Hep3B specific because a prolonged exposure of the cells to this proapoptotic stimulus induces an apoptotic cell death that it is not observed in Hela and HepG2 cells (data not shown). These findings are also reminiscent of the increased sensitivity to environmental stress when yeast strains were deleted of yNatB enzymatic complex (*naa20*-Δ, *naa25*-Δ) [3,4,6].

### Characterization of the in vitro N^α^-acetyltransferase activity of the NatB complex

**Figure 3:**
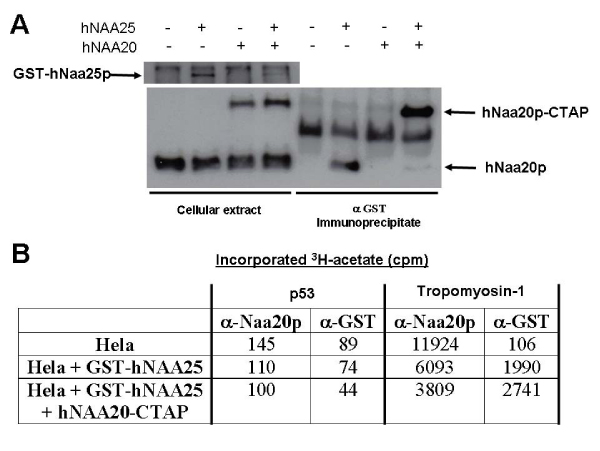
**hNatB purification and enzymatic activity**. A. Coimmunoprecipitation of GST-hNaa25p and hNaa20p-CTAP or GST-hNaa25p and hNaa20p. B. NAT activity of immunoprecipitated hNatB complexes from Hela cells overexpressing GST-hNaa25p and hNaa20p-CTAP with anti-hNaa20p or anti-GST antibodies. Human tropomyosin-1 or p53 aminoterminal peptides synthesized by Dr Francisco Borrás-Cuesta at CIMA University of Navarra as previously described [27] were used as substrates. The results are representative of three independent experiments.

NAT enzymatic complexes are composed by a catalytic subunit and one or several accessory subunits. In the case of hNatA there are two possible catalytic subunits [24] and several accessory subunits [25] but hNatB complex is formed by the catalytic subunit hNaa20p that interacts with hNaa25p to generate an active hNatB complex [5]. We have coexpressed hNaa20p with a carboxi terminal protein tag (CTAP) and hNaa25p fused to GST in Hela cells, detecting an interaction between both fusion proteins (Figure 3A) as it has been described in yeast and human cells before [3-5]. In order to determine if the hNatB enzymatic complex immunopurified with the GST antibodies is enzymatically active we performed an *in vitro *acetyltransferase assay using as substrate a peptide corresponding to human tropomyosin-1 amino terminus, that has been demonstrated to be a hNatB substrate [12]. We observed that immunopurified hNatB complexes, using GST-Naa25p as bait, rescue active enzymatic complexes that acetylate efficiently the tropomyosin-1 aminoterminal peptide, as well as the hNatB complexes immunopurified with a hNaa20p specific antibody (Figure 3B). Thereafter we analyzed if the immunopurified hNatB complexes were able to acetylate p53 aminoterminal peptide, as this protein is a potential NatB substrate based on its first two amino acids (Met-Glu-). Surprisingly, none of the purified hNatB complexes acetylated this peptide.

In order to characterize in more detail hNatB *in vitro *acetyltransferase activity we purified enzymatic complexes with the hNaa20p antibody from Hela cells as this has resulted the most efficient isolation method (Figure 3A). A set of peptides corresponding to the aminoterminal sequences from proteins with a glutamic or aspartic acid after the initial methionine, as those proteins are predicted to be NatB substrates [1,2], were used in the hNatB *in vitro *assay. Only 5 in 19 analyzed peptides were not hNatB acetylated, being good substrates both Met-Asp and Met-Glu- peptides (Table 1). However all the eukaryotic proteins with an aspartic or glutamic acid at position +2 analyzed so far are N-terminaly acetylated [2]. This indicates that the *in vitro *hNatB acetylation activity is not completely reproducing the *in vivo *activity because we are missing some components or characteristics necessary for a perfect hNatB *in vitro *function. But as we are using as substrates 20 amino acid peptides they can lack some structural information necessary for being hNatB substrate as the yeast NatD enzymatic activity [26]. Furthermore, we can not exclude the existence of unknown NATs with an hNatB overlapping substrate specifity in vivo.

**Table 1:** Identification of hNatB *in vitro *substrates.

**PEPTIDE**	**CPM**	**PEPTIDE**	**CPM**
**BAXA**	86	**DCUP**	51
**BRCA1**	384	**KAD1**	502
**CASP8**	457	**B3AT**	72
**CASP9**	7323	**CRBA1**	119
**CDK3**	3489	**PTN1**	2931
**CDK8**	2665	**PPLA**	2741
**FADD**	95		
**TNNC1**	82	No-peptide	74
**1433E**	1763		
**CRYAB**	4077		
**NAT2**	5363		
**MT1A**	1309		
**RS28**	1402		

Immunopurified hNatB complexes with anti-hNaa20p antibody were used to identify 20 amino acids length peptides with Met-Asp- (MD-) or Met-Glu- (ME-) as first two amino acids that are *in vitro *acetylated by hNatB. The results are representative of at least two independent experiments.

In order to prove the relevance of the first two aminoterminal amino acids in hNatB substrates we substituted human tropomosin-1 first, second or both aminoacids by an alanine. As it is observed in Table 2, the replacement of the second amino acid abolishes its nature as hNatB substrate but not the substitution of the first amino acid. Thus the second residue is the most important for determining hNatB substrates and this enzymatic activity is not restricted to methionine acetylation as it can acetylate a different residue such as alanine.

**Table 2:** Characterization of hNatB *in vitro *acetylating sequences.

**PEPTIDE**	**CPM**
**No-peptide**	219
**Tropomyosin-1**	5290
**Tropomyosin-1 MA**	283
**Tropomyosin-1 AD**	1724
**Tropomyosin-1 AA**	221
**β-actin**	4022
**β-actin MA**	3760
**β-actin AD**	2678
**β-actin AA**	2951

To determine the relevance of the first two amino acids there were used tropomyosin-1 and β-actin aminoterminal peptides (20 amino acids long) as well as modifications of these peptides with the first (AD), second (MA) or both (AA) amino acids changed to an alanine in a NAT assay with immunopurified hNatB complexes. The results are representative of at least two independent experiments.

Actin N-terminal processing is very important for a proper actin function [11] being N-terminal acetylation a significant step in this procedure [9,10]. Therefore we extended the analysis to the human β-actin aminoterminal peptide with the same substitutions as the tropomyosin peptide. Surprisingly, all the mutated β-actin peptides were as good substrates as the original sequence (Table 2). Consequently, in some cases there are other amino acids besides the first two aminoterminal that dictate the competence of a peptide as hNatB substrate.

## Conclusion

In conclusion, we have determined that hNatB enzymatic complex is necessary for a proper cell cycle progression and resistance to proapoptotic stimuli in hepatic cell lines as has been observed before in other cell types. hNaa20p downregulation induces cell growth arrest in a p53 dependent and independent manner. hNatB acetylates *in vitro *most of the peptides with Met-Asp- or Met-Glu- amino termini, being more important the aspartic or glutamic acid than the initial methionine for a proper acetylation.

## List of abbreviations used

1433E: 14-3-3 protein epsilon; BAXA: Apoptosis regulator BAX, membrane isoform alpha; BRCA1: Breast cancer type 1 susceptibility protein; CASP8: Caspase-8 precursor; CASP9: Caspase-9 precursor; CDK3: Cell division protein kinase 3; CDK8: Cell division protein kinase 8; CRYAB: Alpha crystallin B chain; FADD: FADD protein (FAS-associating death domain-containing protein); MT1A: Metallothionein-1A; RS28: 40S ribosomal protein S28; TNNC1: Troponin C, slow skeletal and cardiac muscles (TN-C); NAT2: N-acetyltransferase 2 (arylamine N-acetyltransferase); DCUP: Uroporphyrinogen decarboxylase; KAD1: Adenylate kinase isoenzyme 1; B3AT: Band 3 anion transport protein; CRBA1: Beta-crystallin A3; PTN1: Tyrosine-protein phosphatase non-receptor type 1; PPLA: Cardiac phospholamban.

## Competing interests

The authors declare that they have no competing interests.

## Authors' contributions

AA, CG, ML, EL, JP and RA designed research. AA carried out all the experiments concerning cell manipulation and analysis. CG and ML performed the in vitro acetylation assays. All authors designed research and analyzed data. RA coordinated the research and wrote the paper.
